# Cell type and gene regulatory network approaches in the evolution of spiralian biomineralisation

**DOI:** 10.1093/bfgp/elad033

**Published:** 2023-08-17

**Authors:** Victoria A Sleight

**Affiliations:** School of Biological Sciences, University of Aberdeen, Aberdeen, United Kingdom

**Keywords:** biomineralisation, evolution, cell type, gene regulatory networks, Spiralia

## Abstract

Biomineralisation is the process by which living organisms produce hard structures such as shells and bone. There are multiple independent origins of biomineralised skeletons across the tree of life. This review gives a glimpse into the diversity of spiralian biominerals and what they can teach us about the evolution of novelty. It discusses different levels of biological organisation that may be informative to understand the evolution of biomineralisation and considers the relationship between skeletal and non-skeletal biominerals. More specifically, this review explores if cell type and gene regulatory network approaches could enhance our understanding of the evolutionary origins of biomineralisation.

## INTRODUCTION

Most animals use minerals to produce hard structures in a process termed biomineralisation. When we think of biominerals in animals, the first image would likely be a skeleton, or perhaps teeth, but in fact biominerals take a huge diversity of forms and functions including protection, support, dentition, ion storage and even sensing. Biominerals can form on the outside of an animal as shells or plates (exoskeleton), or inside an organism for example as spicules or bones (endoskeleton). Biominerals have diverse functions outside of skeletons. Non-skeletal biominerals include otoliths that are used to sense gravity and acceleration in the vertebrate inner ear or the analogous gravity-sensing statoliths in many invertebrates. Biominerals can also be pathological, they can form in ectopic locations or in excessive amounts, for example, kidney stones or atherosclerosis.

**Figure 1:**
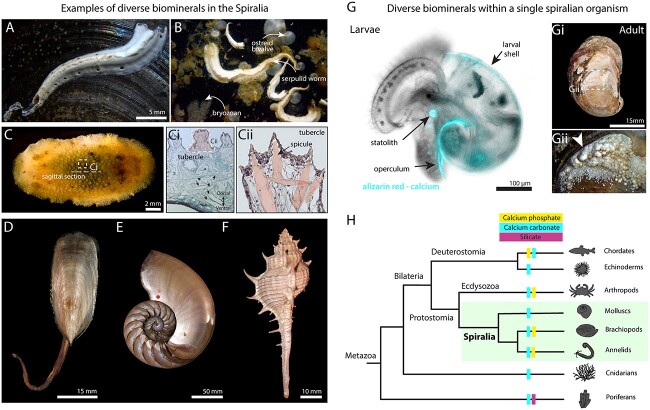
Diverse biominerals. (**A**) A serpulid worm tube on a mytilid bivalve shell. (**B**) A living assemblage of spiralian biomineralisers on settlement panel deployed in the Menai Strait, UK (image supplied by Leyre Villota Nieva, 3°13′34.8”N 4°09′33.8”W). (**C**) Live image of *Doris pseudoargus*, dorsal view. Ci) Sagittal section stained with Masson’s trichrome, examples of spicules highlighted by black arrowheads. Cii) Tubercle spicules zoomed in box from Ci, spicules false coloured coral. (**D**) Brachiopod shell - *Lingula* sp. (BRACH11B). (**E**) Cephalopod shell - *Nautilus pompilius* (ABDUZ100229) F) Gastropod shell – *Murex brevispina* (ABDUZ996). (**G**) *Crepidula fornicata* veliger larval stage live image after staining calcified structures Gi) *C. fornicata* adult shell, dorsal view. Gii) Calcified foot secretions on dorsal side of shell, zoomed in box from Gi. Specimens photographed from University of Aberdeen Museum Collection (ABDUZ100231, ABDUZ100230, ABDUZ100229, ABDUZ996, ABDUZ100229, BRACH11B). (**H**) Schematised phylogenetic tree showing selected examples of the distribution of biominerals across the Metazoa.

Comprising more than one third of extant bilaterian phyla, the Spiralia (segmented worms, flat worms, molluscs, brachiopods and their relatives) is an extremely species rich clade and the range of biomineralisation processes in this group is also impressive (Figure 1). For example, molluscs produce shells made of calcium carbonate (Figure 1A, B, E, F and G) while brachiopods produce shells made of calcium phosphate (Figure 1D). Perhaps the most conspicuous spiralian biominerals are shells, but there are also tubes (Figure 1A and B), stylets, statoliths (Figure 1G), sclerites (or spicules, Figure 1C), plates, love darts and more. In addition, structures such as the molluscan and annelid operculum and molluscan foot can be mineralised. Even within a single organism there can be a range of skeletal and non-skeletal biomineralised structures, for example, an individual gastropod could have a mineralised shell (larval and adult), statolith, operculum and foot (Figure 1G). The relative homology of biomineralised structures within a spiralian organism is not fully understood [1].

Biomineralisation is a fundamentally important process across the Spiralia. Skeletal biomineralisation in the form of shells, spicules and plates are structural components of body plans and so skeletal biomineralisation is likely critically important in morphological evolution [2]. Meanwhile non-skeletal biomineralisation is physiologically important in sensing the environment [3], immunity [4] and even reproduction in some species [5]. Spiralians are epitomised by diversity on multiple evolutionarily important levels, they include a fantastic range of body plans and extensive examples of novelty. The multiple independent emergences of biomineralisation in the Spiralia is an excellent system to examine evolutionary novelty. Major unanswered questions include where did biomineralisation come from each time it evolved in the Spiralia? Was a gene regulatory network (GRN), required to develop and maintain cells and tissues that biomineralise, independently co-opted from existing GRNs? Or has evolution taken different routes to a convergent biomaterial? If homology does exist between spiralian biominerals, what level is it at (GRN, cell type, embryonic origin, morphology)?

This review will consider various levels of biological organisation that are informative to understand the evolution of biomineralisation, taking the view that homology can exist at different independent levels [6]. More specifically, it will explore if cell type and cell biology approaches could enhance our understanding of the evolutionary origins of biomineralisation, drawing attention to the extensive diversity of biominerals in spiralians and what unique answers they could bring to the big questions in biomineral evolution. Previous reviews have provided in-depth syntheses of metazoan, spiralian or specific phylum biomineralisation from various perspectives including: early drivers of animal biomineralisation [7], evolutionary history [2, 8–10], mineralogy [11, 12], developmental biology [13, 14], ion transport pathways [15, 16] and more. Here I will briefly cover some of the leading hypotheses and patterns in animal biomineralisation to give context to the evolution of spiralian biomineralisation. I will then discuss the cell types that produce biominerals and consider the role of non-skeletal biominerals as possible evolutionary pre-cursors to biomineralised skeletons. Much of the themes covered in the review stem from a question many in the field of biomineralisation have likely pondered: what exactly makes a cell capable of biomineralisation?

### Multiple independent origins of biomineralised skeletons

#### The earliest metazoan biomineralised skeletons

Animals began making biomineralised skeletons hundreds of millions of years ago. In the late Ediacaran, around 550 million years ago (Mya), widespread animal skeletons appeared in the fossil record. Of those initial metazoan biomineralisers, *Cloudina* is likely the earliest skeleton forming animal, it built a skeleton made of nested calcite funnel-like structures and has been found in sedimentary rocks worldwide [17, 18]. Over the following 10–20 million years the Cambrian Explosion took place, all major phyla emerged and at the same time, widespread mineralised skeletons were innovated (Figure 1H). Some researchers focus on the question of what ‘triggered’ animal biomineralisation in the Ediacaran-Cambrian transition. Answering this question would help to unravel the mystery of why widespread biominerals suddenly appear in the fossil record billions of years after life began [19, 20]. Whatever the causative factors, environmental or biological, the current consensus is that biomineralised skeletons in animals are an evolutionary novelty that emerged multiple times independently [2, 8, 9].

A range of evidence supports the hypothesis that animals have evolved biomineralised skeletons multiple times independently over a relatively short period of time in the early Cambrian (circa 25 million years, [2]). This evidence has been explored deeply in recent work and so I will only briefly summarise for context here [8, 9]. In the fossil record, there doesn’t appear to be a phylogenetically clustered organisation to the first appearance of biomineralised skeletons, and this is generally interpreted as skeletons evolving independently in different biomineralising lineages [9]. In addition, taxa that possess biomineralised skeletons are pre-dated in the fossil record by soft-bodied representatives that lack a biomineralised skeleton [21–23]. The interpretation here is that major phyla and body plans had already diverged prior to the evolution of biomineralised skeletons, the last common ancestor to these phyla was therefore entirely soft-bodied and biomineralised skeletons evolved independently in each taxa [8, 9, 11]. Another factor authors have considered is the relative ‘biological control’ exerted in the production of biominerals. In microbial mineralisation there is a well-accepted and defined term ‘organomineralisation’ that encompasses two types of biomineral production: biologically induced and biologically influenced mineralisation [24]. The former is intrinsic and a by-product of metabolic processes and the latter is extrinsic, environmentally driven and entirely passive. In eukaryotic biomineralisation however, there are not strict definitions relating to the level of biological control exerted in biomineralisation. Instead authors have described simple, non-heirarchical three dimensional organisations in mineralogy, such as fibrous or microgranular microstructures, as ‘loosely’ biologically controlled [9]. More complex, multi-layered and hierarchical microstructures, such as nacre, have been described as ‘tightly’ controlled [9, 19], implicitly suggesting there may be a continuum of biological control exerted over the composition, microstructure and morphology of biominerals. A proposed prediction of multiple independent origins of biomineralised skeletons is that a high diversity of simple and loosely biologically controlled biominerals would precede increasingly complex and tightly controlled ones, and that as biological control increased, variation within specific lineages would decrease, as selection pressure ‘fixes’ biomineralising pathways in each taxa. This pattern of highly diverse loose biological control to more fixed and complex microstructures, appears to hold true for biomineralised skeletons across the animal tree of life [9, 19], but an opportunity awaits for those who can precisely define biological control and accurately quantify its variance in the fossil record and extant taxa.

#### Convergent evolution of metazoan biomineralisation

The calcium carbonate biomineralised skeletons of marine organisms are thought to be an example of convergent evolution that can be described in a single integrated model [8, 11]. In this convergent evolution scenario, it is hypothesised that multiple independent evolutionary routes have given rise to an identical biomineralisation process, or framework. This model has the assumption that biomineralisation occurs in a privileged space that is partially open but chemically different to seawater. The first step in the model is endocytosis of either calcifying fluid or seawater by tissues at the margin of the privileged space. The endocytosed fluid is then enriched in calcium and carbonate using ion membrane transporters, and protons are removed to increase pH. This endocytosed fluid then becomes amorphous calcium carbonate (ACC). ACC-H_2_O solid particles are then exocytosed into the privileged space. Particles and ions attach to the growing front of the biomineral and remain ACC, before crystalising into crystalline calcium carbonate. Currently however, empirical data supporting the physiological existence of the model has been observed directly in a single deuterostome (urchin, [15]), and indirectly in a cnidarian (coral, [25]) – both examples are in groups of endoskeleton producing organisms. To understand if this model is evolutionarily informative for metazoan biomineralisation much work is needed to address if it applies to all biomineralising skeletons, especially those with exoskeletons. The cell biology and genetic control of each step in the model needs to be validated across the tree of life.

#### Spiralian biomineralisation

**Figure 2:**
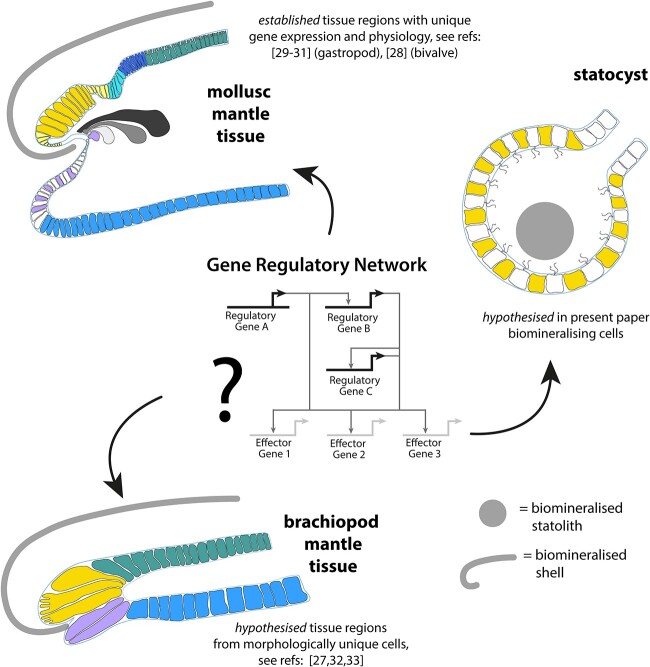
Schematised overview of selected hypothesised biomineralising cell types in spiralians, highlighting unknowns in the GRNs that define them. Previously described cell populations are based on morphology, histology, ultrastructure and candidate gene expression of tissue regions and are differentiated using colours. Biomineralising tissue regions have not yet been characterised to cell type resolution in any spiralian. The same colours between groups represent a framework and hypothetical examples of cell type homology, or GRN homology, there is no current data supporting this. Hence, it is a priority to define biomineralising cell types and the GRNs that produce them in the Spiralia. Schematised drawings adapted after [27–33]. Illustrations modified under a CCBY4.0 license (https://creativecommons.org/licenses/by/4.0/).

The Spiralia is an excellent system to study the evolution of biomineralisation. This group contains around 40% of all metazoan phyla (14/36) including some of the most iconic examples of biominerals. Spiralian skeletons are a combination of primarily exoskeletal shells and plates, with some groups also having endoskeletal spicules (for example, molluscs). The exact phylogenetic relationships among the Spiralia are not fully resolved but almost all share a highly conserved early mode of development: spiral cleavage [26]. Similar to the difficulties in resolving the phylogenetic relationships among spiralians, it is also difficult to be sure on the evolutionary route to biomineralisation in these groups. Most work has focussed on brachiopods and molluscs and taken either a gene regulation or palaeontology perspective. To understand the dynamic process of gene regulation we typically use a network framework (Figure 2). Gene regulatory networks (GRN) are a way to understand hierarchical logic and complex interactions between genes and their products. Upstream components of a GRN are typically transcription factors, they often activate genes involved in signalling cascades that eventually switch-on the expression of downstream effectors. Taking the GRN approach, some authors hypothesise that biomineralisation is ancestral to molluscs and brachiopods (either calcium carbonate or calcium phosphate) [34]. Using evidence such as the spatial expression of transcription factors and downstream effectors (but not gene function tests or cis-regulation), recent work suggests that the common ancestor of molluscs and brachiopods biomineralised in some capacity and that at least some nodes in the biomineralising GRN have been partially retained in both lineages. It is hypothesised that the nodes partially retained from a biomineralising GRN have then been independently co-opted into shell development and formation [27]. Other authors, however, argue that an unmineralised common ancestor and multiple origins of biomineralisation in the molluscs makes it most likely that the common ancestor to brachiopods and molluscs had unmineralised chitinous structures that were inherited and independently mineralised in each lineage [9].

Studying the molecular control of biomineralisation in the Spiralia to understand its evolutionary origins has so far centred around two observations: (1) there is a small set of ‘core’ biomineralisation genes that seem to be present in all biomineralising groups, which could represent components of an ancient GRN that have been repeatedly co-opted [9, 34–41]. Conversely, (2) most biomineralising genes are rapidly evolving including a high proportion of lineage-specific genes [40, 42–44]. A huge amount of research effort has produced tissue-specific transcriptomes and proteomes of biomineralised skeletons in the Spiralia, especially in molluscs [28, 45–54]. The nature of these bulk transcriptomic and proteomic approaches is that downstream effectors, such as biomineralisation enzymes and extracellular matrix proteins, have been extensively identified and characterised as they are detectable and highly expressed in these samples. Genes that are typically lowly expressed, such as regulatory upstream transcription factors and signalling molecules, however, have been more challenging to study.

A consensus biomineralisation GRN for any representative of the Spiralia is lacking, but computational approaches have been used to make GRN predictions [55]. The observations of a small number of ‘core’ biomineralisation genes versus a large number of rapidly evolving, lineage-specific genes are therefore, currently, only generalisable to the downstream effectors in biomineralisation. In addition, most research on the molecular control of spiralian biomineralisation has focussed on adult tissues. Skeletogenesis in developmental stages has received less focus. In molluscs the early larval shell is phenotypically more conserved than that of adults and so we may expect there to be more conservation at the molecular level in developmental stages. Surprisingly though, the two general observations of a handful of ‘core’ genes versus a large number of rapidly evolving and lineage-specific genes appears to hold true, at least in the downstream effectors in bivalve larval skeletogenesis [44]. In addition, not only are there a large number of lineage-specific genes in larval skeletogenesis, it also appears that the genes used to build the larval shell are almost entirely different to that of the adult shell [44, 56, 57]. These observations apply mainly to downstream effectors, it is possible however that a largely conserved GRN could be driving diverse downstream effectors in different life history contexts, with only small tweaks to the upstream GRN. This phenomenon in morphological evolution has been revealed in remarkable detail in *Drosophila* [58].

Biomineralised skeletons in the Spiralia, and more generally in animals, have evolved multiple times, but the question remains: how? Or perhaps more precisely: where from? Yes, biomineralised skeletons are likely independently evolved, and a clear example of a complex novel phenotype that has repeatedly evolved in different lineages. In each of the independent evolutionary events though, what precise mechanism(s) facilitated that evolutionary transition, and to what extent is there bona fide de novo novelty versus co-option or modification of more deeply homologous pre-cursors [59, 60]? The examples briefly explored above give some context to the vast evolutionary distances spanned when considering the evolution of biomineralised skeletons, for example corals and urchins last shared a common ancestor around 700 Mya and many biomineralised spiralian skeletons first appear in the fossil record in the Cambrian period between approximately 540–500 Mya [8]. Evidently the task of reconstructing genetic, cellular, and developmental events that happened so long ago, is far from trivial. An integration of expertise is required from palaeontology, biophysics and mineralogy, as well as developmental, molecular and cellular biology.

### Spiralians can uncover how biomineralising cell types evolve

Cells are the building blocks of all metazoans, they are studied in huge detail as functional units of biology [61–63]. Most recently, technical gains such as single-cell resolution sequencing and connectome resolution volume electron microscopy have facilitated the rapid description and evolutionary comparison of many cell types. In terms of evolutionary biology, these gains have been particularly transformative in non-traditional model systems, in organisms at essential phylogenetic nodes on the tree of life [64–67]. Cells can be grouped into types based on a variety of different traits for example, morphology, ultrastructure, function, gene expression, developmental trajectory, etc. The term ‘cell type’ therefore, has many different definitions [68]. Here, for thinking about the evolution of biomineralisation, an evolutionary definition is appropriate. Arendt *et al.* define a cell type as: ‘a set of cells in an organism that change in evolution together, partially independent of other cells, and are evolutionarily more closely related to each other than to other cells. That is, cell types are evolutionary units with the potential for independent evolutionary change’ [69].

Given the hypothesised multiple independent origins of biomineralisation in the Spiralia, the cell type prediction is that there are also many evolutionarily independent biomineralising cell types in spiralians. One example of biomineralising cell types are the cells in the mantle tissue of molluscs that make the shell. Mantle tissues were histologically and ultrastructurally described in the twentieth century [31, 70, 71]. More recently, spatial localisation of mRNA using *in situ* hybridisation techniques has revealed what is described as ‘cellular heterogeneity’, ‘cell populations’ and ‘mantle modularity’ at the molecular gene expression level in both developmental and adult stages (Figure 2 [28–30, 72, 73]). For example, in the pond snail *Lymnaea stagnalis,* a shell matrix encoding gene *sfc 22* with sequence similarity to Pif97 is specifically expressed in the outer epithelium of ‘zone 5’ in the adult mantle tissue [30]. In the bivalve *Laternula elliptica, pif* expression is restricted specifically to the outer epithelium on the outer fold of the mantle edge [28]. An intriguingly similar morphological cellular arrangement is seen in the biomineralising mantle tissue of brachiopods where it is thought that within the outer mantle epithelium lobate cells secrete the organic portion of the shell and vesicular cells secrete the inorganic portion of the shell (Figure 2 [74–76]). Unlike in molluscs however, gene expression has not yet been spatially localised to these morphologically distinct cells in the brachiopod mantle. Much work is ahead in spiralians to identify which cell populations are true cell types (as per an evolutionary definition) that are functionally involved in biomineralisation, and the GRNs that produce them (Figure 2). Only once these cell types and GRNs have been defined can we test hypotheses of homology at different levels (morphological structure, cell type or GRN) through systematic comparison within a rigorous phylogenetic framework (Figure 2).

Most work that discusses the evolution of biomineralisation, is implicitly referring to the evolution of biomineralised skeletons. These large structures, such as bones and shells, make up most of the fossil record. Palaeontology is fundamental to understand the evolution of diverse forms of life. The gold standard is a set of confidently dated, excellently preserved, transitional fossils that document the gradual transition from one form to another, a beautiful example is the fin to limb transition in vertebrate evolution [77]. The evolution of very small structures however, is more difficult to piece together from fossils. For example, many spiralian larval stages are very small (<1 mm in diameter), and have even smaller biominerals such as statoliths, which are calcified stones within gravity sensing organs (around 0.01 mm in diameter [78]). Statoliths persist into adulthood in some groups where they remain small (around 0.01 mm – 0.15 mm in diameter [79]). Micro-biominerals, such as statoliths, are largely neglected in spiralian palaeontology, except perhaps in cephalopods where their distinct morphology has been useful in identifying fossils [80–82]. Due to the difficulty in identifying very small non-skeletal biominerals in the fossil record it is difficult to know if non-skeletal biomineralisation played a role in the evolution of skeletons we study today. Could non-skeletal biominerals, generated using a deeply ancient GRN, be an evolutionary precursor to biomineralised skeletons? Given that the fossil record is unlikely to be able to shed like on this question, one approach to elucidate the earliest origins of biominerals is to turn to developmental and cellular biology and ask: ‘are there homologies in the GRNs that produce skeletal and non-skeletal biomineralising cells?’ (Figure 2).

Returning to the evolutionary origins of biomineralised skeletons in animals, work in taxa with a richer history in cell biology has characterised biomineralising cell types using many different traits. In deuterostomes for example, osteoblasts in vertebrates and primary mesenchymal cells in echinoderms have been studied in detail. One of the most well-defined GRNs is that of urchin skeletogeneisis and the specification of larval skeleton producing primary mesenchyme cells [83]. Recently, using a GRN approach, it has been hypothesised that sea urchin spiculogenesis evolved via independent co-option from an ancestral VEGF-signalling based tubulogenesis programme [84]. The work leading to the VEGF co-option hypothesis was vast, involving decades of focussed research from multiple large research groups. The same focus on cell biology and elucidating GRNs has not previously been tractable in spiralians. The advent of single cell sequencing technologies however, is likely to rapidly accelerate our ability to define and reconstruct the evolutionary history of cell types in this group. Early candidate gene approaches have identified transcriptions factors and signalling pathways that are likely to be important. Signalling pathways that have been implicated in skeleogenesis and biomineralisation in spiralians include: BMP/DPP [85–90], WNT [44, 91], TGF-β [1, 88] and Dopamine [92]. And transcription factors include: *engrailed* [93], *pou3f4* [94], *grainyhead* [1], *hox1* [95, 96], *hox 4* [95, 96], *post1* [96], *post2* [96], *gata2/3* [97], *soxc* [98], *pax2/5/8* [72]. Candidate genes such as these will be the starting point to define biomineralising cell types and GRNs from single-cell datasets, but ultimately gene function and regulation studies will be necessary to validate gene interaction at key nodes in the GRN.

### Summary

This review has provided a glimpse into the fantastic diversity of biominerals in spiralians both within and between groups (Figure 1), this diversity offers an excellent system to study multiple independent evolutionary origins of novel complex phenotypes. To date, spiralian biomineralisation research has focussed heavily on adult tissues and downstream effectors such as enzymes and extracellular matrix proteins, but it may be more evolutionarily informative to study upstream components of the GRN. I offer a hypothesis that micro-biominerals, such as statoliths, may have been an important stepping-stone in acquiring the ability to produce a biomineralised skeleton. Studying organisms that produce multiple different biomineralised structures and asking questions about cell types, GRNs and their homologies will be required to test such hypotheses (Figure 2). Advances in technology gives the spiralian community a key to unlocking the integrative GRN and cell type approach required to uncover the evolutionary origins of biomineralisation in this important group.

**Key Points**

- Animals have evolved the ability to produce biominerals multiple times independently
- Spiralians have a huge diversity of skeletal and non-skeletal biominerals
- There are many open questions in the evolution of biomineralisation and an integrated approach covering a diversity of taxonomic groups is required to tackle them
- Precisely defining the cell types and gene regulatory networks that produce biominerals in spiralians is a priority
- Non-skeletal biomineralisation could have been an evolutionary precursor to skeletal biomineralisation in the Spiralia

## Notes

**Victoria A. Sleight** has a long-standing interest in biomineralisation and skeletal systems. She completed her PhD at the British Antarctic Survey, where she studied biomineralisation in bivalves, and her postdoc at the University of Cambridge focussing on body plan evo-devo in the little skate. Victoria now runs a research group at the University of Aberdeen focussing on the evolutionary development of skeletons, primarily in marine invertebrates.
